# Mutation in *PHKA2* leading to childhood glycogen storage disease type IXa: A case report and literature review

**DOI:** 10.1097/MD.0000000000017775

**Published:** 2019-11-15

**Authors:** Qian Zhu, Xiao-Yu Wen, Ming-Yuan Zhang, Qing-Long Jin, Jun-Qi Niu

**Affiliations:** Department of Hepatology, The First Hospital of Jilin University, Changchun, China.

**Keywords:** case reports, genetic diseases, glycogen storage disease, hepatomegaly, mutation, X-linked

## Abstract

**Introduction:**

Glycogen storage disease (GSD) type IX, characterized by liver enlargement and elevated aminotransferase levels, is the most frequent type of GSD. The global incidence of GSD type IXa is only about 1/100,000 individuals. Case reports of GSD type IX are rare in China. We present the first case report of GSD type IXa in Northeast China caused by mutation of *PHKA2*.

**Patient concerns:**

An 11-year-old boy was referred to our hospital because of liver enlargement with consistently elevated transaminase levels over 6 months.

**Diagnosis:**

Histopathological results following an ultrasound-guided liver biopsy confirmed a diagnosis of GSD. Further genetic testing showed that the patient had GSD type IXa caused by the c.133C>T mutation in *PHAK2*.

**Interventions:**

We placed the patient on a high-protein and high-starch diet and provided hepatoprotective and supportive therapy.

**Outcomes:**

The patient's transaminase levels decreased significantly and were nearly normal at 10-month follow-up.

**Conclusion:**

This is the first reported case of GSD type IXa in Northeast China. We hope that the detailed and complete report of this case will provide a reference for the diagnosis of liver enlargement of unknown etiology in future clinical practice.

## 1 Introduction

Glycogen storage disease (GSD) types I, III, IV, VI, and IX are generally related to liver dysfunction. The most frequent subtype, GSD type IX, is caused by phosphorylase kinase (PhK) deficiency and accounts for 25% of all GSD.^[1]^ The global incidence of GSD type IX is only about 1/100,000^[1]^ individuals. Case reports of GSD type IXa are rare in China. Until now, there were no reports of GSD type IXa occurring in Northeast China. We present the first case diagnosed in our hospital of a child with GSD type IXa, which was caused by a mutation in *PHKA*.

## 2 Case presentation

A 11-year-old boy was referred to our hospital for liver enlargement with significantly elevated transaminase levels.

### 2.1 History

The patient went to a local hospital after experiencing nausea for no obvious cause for a period of 6 months. Examinations revealed liver enlargement and elevated transaminase levels. The patient subsequently took Chinese medicinal herbs (uncertain dose and composition) for 2 months, and his symptoms improved. A follow-up examination showed that the liver was still enlarged. The patient's chief complaint at that time was slight upper abdominal discomfort on the right side. The patient was then received by our hospital for further diagnosis and treatment. The patient was healthy before experiencing his current symptoms, and factors that might cause liver injury, including infection, autoimmune disease, alcohol consumption, or ingestion of other chemical drugs and poisons, were excluded.

### 2.2 Physical examination

**Figure 1:**
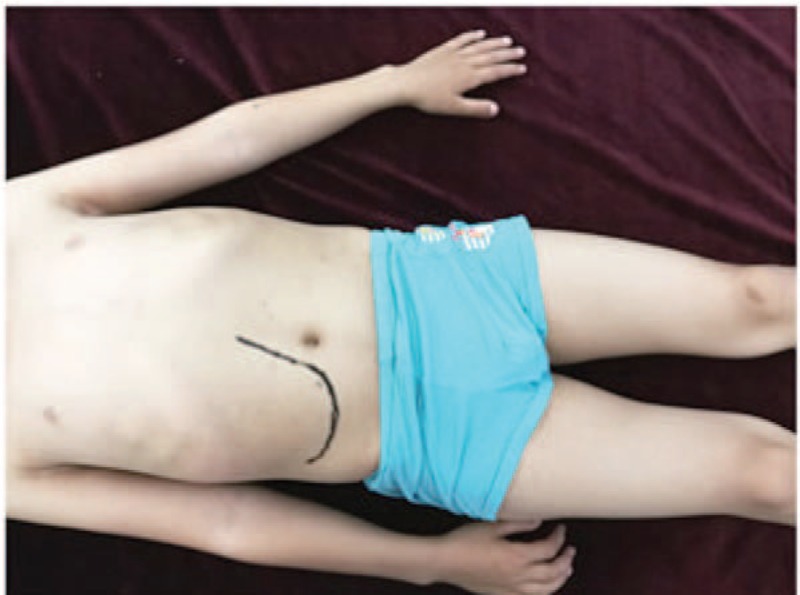
Abdominal physical examination: superficial abdominal veins could be exposed, and palpation revealed the liver edge (black lines) 8 cm below the costal margin and 7 cm below the xiphoid with normal texture, smooth surface, and sharp edge. The spleen was not palpable.

The patient was 130 cm tall, weighed 31 kg, and displayed dysplasia of the teeth and abnormal relaxation of the ligament of the metacarpophalangeal joint. The superficial abdominal veins could be exposed by palpitation, and the liver edge could be felt 8 cm below the costal margin and 7 cm below the xiphoid (Fig. 1).

### 2.3 Investigations

**Figures 2:**
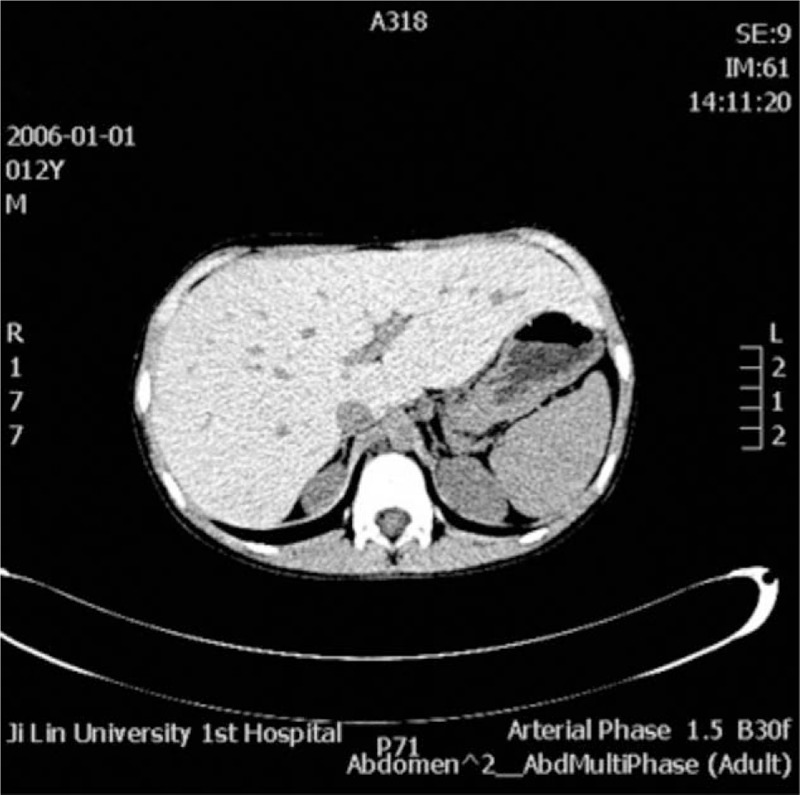
Abdominal noncontrast computed tomography (CT) scan showed marked enlargement of the liver.

**Figures 3:**
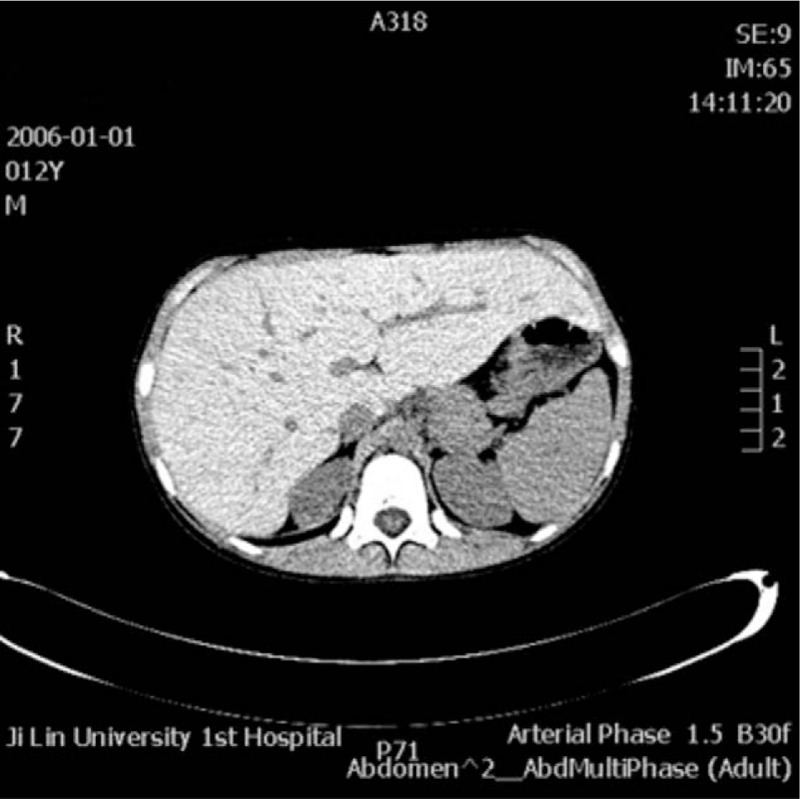
Abdominal noncontrast computed tomography (CT) scan showed marked enlargement of the liver.

**Figure 4:**
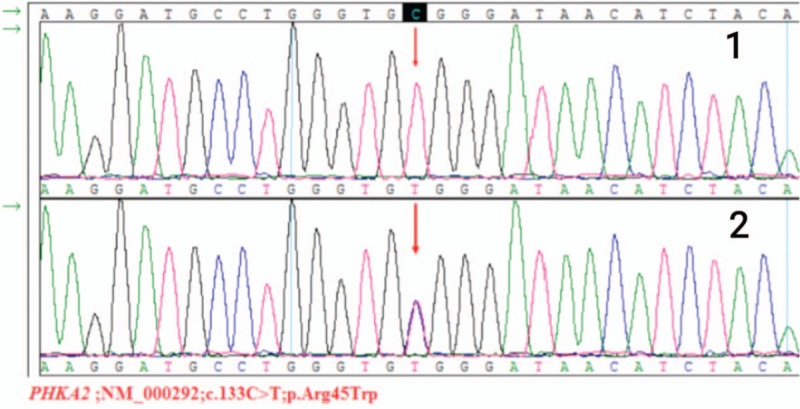
Sanger validation of the c.133C>T (p.Arg45Trp) mutation in *PHKA2* identified by whole-genome sequencing: the proband is hemizygotic and the proband's mother is heterozygotic.

**Figure 5:**
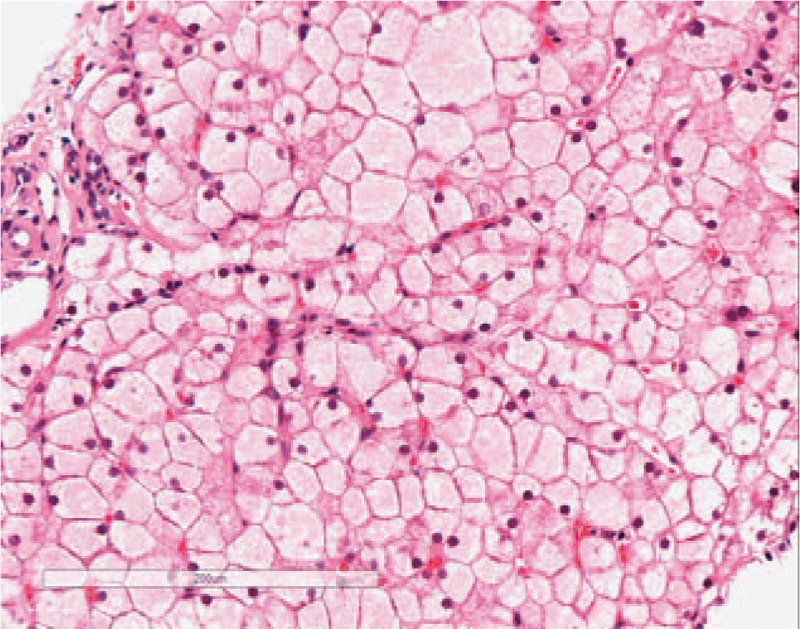
H&E staining under 200× magnification: hepatocytes appear swollen with light red cytoplasm and small nuclei, closely resembling plant cells. Hepatic sinusoids appear with or without stenosis, presenting a mosaic image.

**Figure 6:**
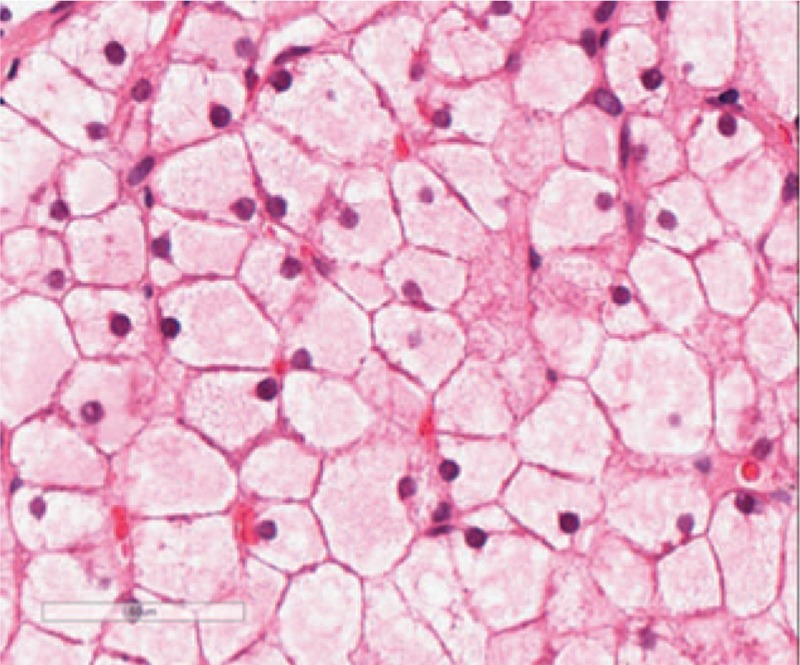
H&E staining under 400× magnification: hepatocytes appear swollen with light red cytoplasm and small nuclei, closely resembling plant cells. Hepatic sinusoids appear with or without stenosis, presenting a mosaic image.

**Figure 7:**
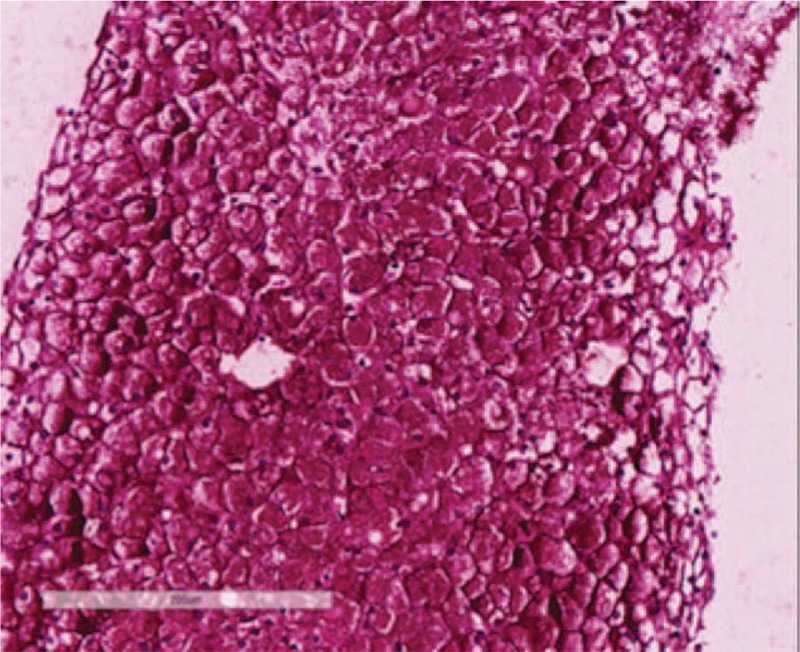
PAS staining under 200× magnification: hepatocytes contain a large amount of positive staining.

**Figure 8:**
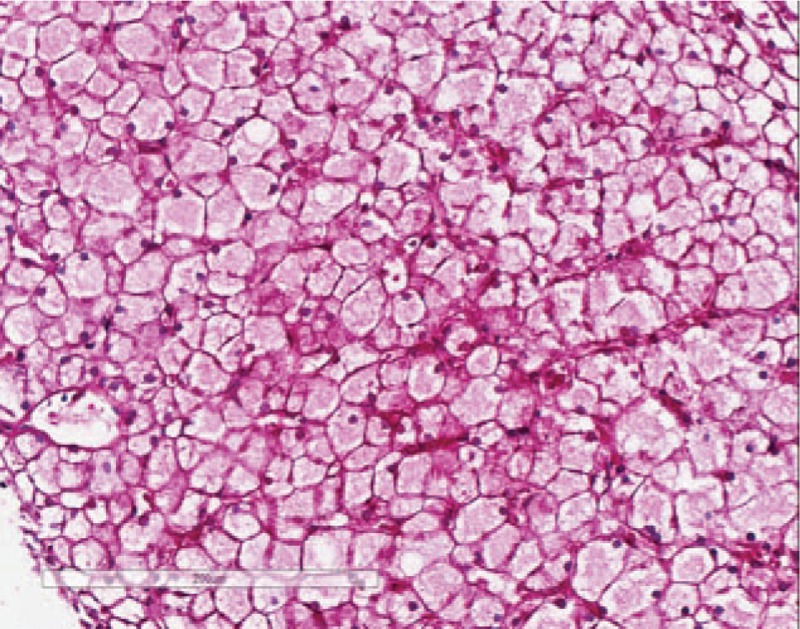
D-PAS staining under 200× magnification: periodic acid–Schiff (PAS)-positive substances are intolerant to the digesting of amylase.

Blood tests confirmed that the patient had elevated aminotransferase levels (aspartate aminotransferase 77 U/L, alanine aminotransferase 102.7 U/L). An abdominal noncontrast computed tomography (CT) scan was performed, which showed only a marked enlargement of the liver (Figs. 2 and 3). We tested for infectious factors that might cause liver injury, including hepatitis virus, Epstein-Barr virus, cytomegalovirus, and parasites, and found no signs of infection. Tests for autoimmune disease-related antibodies conducted at follow-up examinations were negative. On the basis of the patient's age, history, symptoms, and test results, we suspected a genetic or metabolic disease. We screened for abnormal copper and iron metabolism, but the related biomarkers were all normal. During the patient's stay in our hospital, we noted a slightly low fasting blood glucose level, which was still within the normal range (3.69 mmol/L), and a high blood triglyceride level (2.17 mmol/L). Repeated tests produced the same results of high triglyceride level and slightly low glucose level, which suggested abnormal glucose metabolism and the possibility that the patient had GSD. After obtaining permission from the patient's parents, we performed an ultrasound-guided liver biopsy. Histopathological examination of the biopsy sample with periodic acid-Schiff staining showed massive accumulation of glycogen in the hepatic tissue (Figs. 5–8), leading to a final diagnosis of GSD. With further consent from the patient and the patient's mother, we performed whole-genome sequencing on the patient and the patent's mother to get the exact genotype underlying the GSD. Analysis of the patient's genetic sequences revealed a mutation known to cause GSD; the cytosine at nucleotide position 133 in the *PHKA2* gene on the X-chromosome was replaced by thymidine (c.133C>T), resulting in a change from arginine (Arg) to tryptophan (Trp) at amino acid position 45 in the PhK protein. The patient's mother was also found to carry the c.133C>T mutation in *PHKA2*, indicating that the patient inherited the mutation from his mother (Fig. 4).

### 2.4 Treatment

We placed the patient on a high-protein, high-starch diet and provided hepatoprotective and supportive therapy. The patient attended regular follow-up visits with careful re-examinations from June 2018 until March 2019.

## 3 Literature review

In order to roughly evaluate the morbidity of GSD type IXa, we did a literature search in May 2019. We systematically identified all potentially relevant case reports in the past 20 years (2000–2019) in Asia from 3 electronic databases: MEDLINE, PubMed, and Web of Science. Search terms such as “glycogen storage disease type IXa,” “glycogen storage disease,” “PHKA2 gene,” “case report,” and “Asia” were used in various combinations and permutations across the databases. Finally we made screening for 36 cases with detailed history information.

We individually analyzed data of the 36 patients including age, region, the site of mutation, and clinical manifestation (Table 1^[11–18]^). The onset of GSD type IXa can be in young age groups and the average age is 7.2 years. In the total 36 cases, 23 cases from China, accounting for 63.8% of the total 36 cases, followed by the Korea (22.2%).The proportion of cases from Japan (13.9%) is the lowest. It is noteworthy that the most patients in the table we mentioned were mainly manifested as liver enlargement (91.60%) and elevated transaminase (94.40%) in the early stage. However, less than half of the 36 patients showed signs of hypoglycemia (47.20%), hyperlipidemia (30.50%), and short stature (38.80%).

**Table 1:**
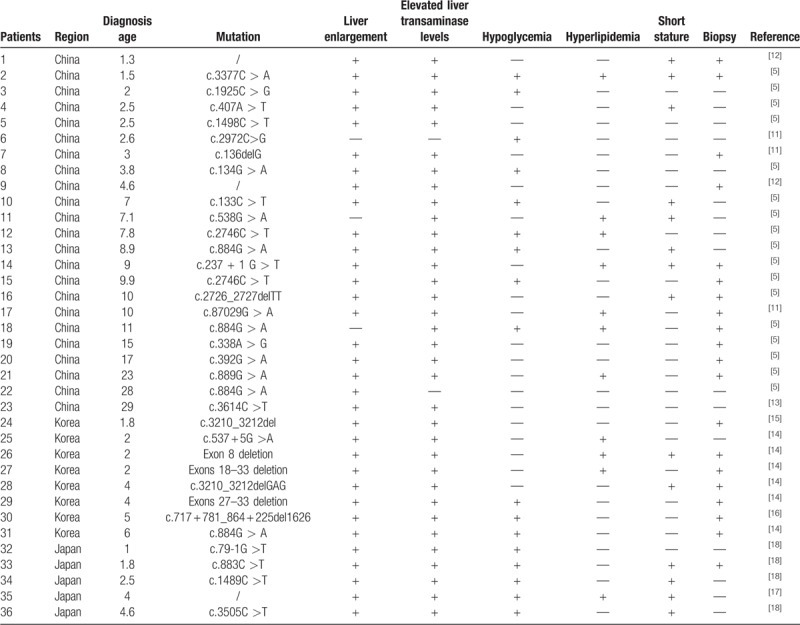
Summary of the clinical data of the 36 patients with glycogen storage disease type IXa in the past 20 years (2000–2019) in Asia.

## 4 Discussion

GSD is a group of genetic and metabolic disorders caused by various enzyme deficiencies. Deficiency of PhK causes GSD type IX. The first case report in the literature of PhK deficiency was in 1966.^[2]^ PhK plays a role in blood glucose regulation by catalyzing the activation of glycogen phosphorylase. The PhK protein consists of 4 subunits, denoted α, β, γ, and δ, each of which is encoded by a separate gene. GSD type IX is thus classified into 4 subtypes depending on which gene is mutated.^[3]^ The *PHKA2* gene, located on the long arm of the X-chromosome (Xp22.2–22.1) encodes the α subunit, which includes 33 exons. Mutation of *PHKA2* results in GSD type IXa, also known as X-linked liver GSD, which accounts for approximately 75% of all cases of GSD type IX.^[4]^ About 134 cases of GSD type IXa have been reported at present, and 99 types of *PHKA2* gene mutation have been documented in the Human Gene Mutation Database, including 47 missense mutations, 28 deletion mutations, 9 insertion mutations, 9 nonsense mutations, and 6 splice-site mutations.^[5]^ Most patients with GSD type IXa are characterized by an enlarged liver and increased aminotransferase levels. In addition, some patients exhibit delayed growth. Compared with other types of GSD, GSD type IXa are generally mild, with many patients being asymptomatic or only experiencing a mild increase in blood triglyceride level. Hypoglycemia and hyperlactacidemia are less frequent symptoms of GSD type IXa.^[6]^ Because of its generally mild, atypical symptoms, GSD type IXa difficult to diagnose and is rarely reported in China. The related biomarkers, such as blood glucose and aminotransferase levels, are often changed very little, so they may be ignored or given little concern. Although liver biopsy can provide an exact diagnosis of GSD, it is invasive and cannot provide information about the exact type of GSD, so it has little prognostic value. Furthermore, liver biopsy provides no information about other family members. For those reasons, liver biopsy is often unacceptable to patients and parents of children. On the contrary, genetic testing is the most objective, secure, and reliable method. Genetic testing provides the exact type of GSD and therefore also has prognostic value. There is still progress to be made with genetic testing for GSD, however, because the procedure is complex and relative expensive. It is our hope that with further advancements liver biopsy can be gradually replaced by genetic testing for GSD. GSD type IXa is commonly considered a kind of benign lesion. Long-term follow-up studies of patients with GSD type IXa have shown that the clinical symptoms, including liver enlargement and increased aminotransferase levels, can gradually disappear, and the growth, which is delayed early on, may achieve a standard state in the later stages and even tend to be normal in adulthood.^[7]^ Not all cases have such favorable outcomes, however, and some even progress to liver cirrhosis.^[8]^ Although some specialists view GSD type IXa as self-healing and asymptomatic, the frequent morning nausea and vomiting can still have a negative impact on children's social and academic development and quality of life. Furthermore, the psychological effects caused by growth delay and shortened stature during adolescence should not be ignored.^[9]^ In addition, studies have shown that treatment and management of the glycogen metabolic disorder caused by GSD can remarkably improve the clinical symptoms and biomarkers.^[10]^ Hence, early treatment with a high-starch and high-protein diet and involvement in long-term follow-up with ultrasound examinations to monitor liver function and relief of symptoms can prevent complications and improve patients’ quality of life.

The patient in this case report was mainly characterized by liver enlargement and elevated transaminase levels. Because of the mild clinical symptoms, more detailed laboratory tests should be considered for such patients. In this case, laboratory tests revealed low blood glycogen and high triglyceride levels, which caused us to suspect GSD. This is the first reported case of GSD type IXa in the area of Northeast China. We hope that the detailed and complete report of this case will provide a reference for the diagnosis of liver enlargement of unknown cause in future clinical practice. There are some limitations to this single case study. For privacy reasons, only the patient and the patient's mother could be genetically tested. It would be useful to perform genetic screening of the patient's entire family and to establish a long-term follow-up to monitor the patient's progress.

## Acknowledgments

The authors thank their patient and his guardian for giving informed consent for publication of the case.

## Author contributions

**Conceptualization:** Jun-Qi Niu.

**Data curation:** Qian Zhu.

**Formal analysis:** Xiao-Yu Wen.

**Funding acquisition:** Jun-Qi Niu.

**Investigation:** Xiao-Yu Wen.

**Methodology:** Xiao-Yu Wen.

**Resources:** Ming-Yuan Zhang.

**Software:** Ming-Yuan Zhang.

**Supervision:** Qing-Long Jin.

**Validation:** Qing-Long Jin.

**Writing – original draft:** Qian Zhu.

**Writing – review and editing:** Xiao-Yu Wen.
